# Prehospital identification of trauma patients with early acute coagulopathy and massive bleeding: results of a prospective non-interventional clinical trial evaluating the Trauma Induced Coagulopathy Clinical Score (TICCS)

**DOI:** 10.1186/s13054-014-0648-0

**Published:** 2014-11-26

**Authors:** Martin L Tonglet, Jean Marc Minon, Laurence Seidel, Jean Louis Poplavsky, Michel Vergnion

**Affiliations:** Emergency Medicine, Trauma and Bleeding Care, CHR de la Citadelle, boulevard du 12eme de ligne, 1, 4000 Liège, Belgium; Laboratory and Blood Transfusion Departments, CHR de la Citadelle, boulevard du 12eme de ligne, 1, 4000 Liège, Belgium; CHU du Sart Tilman, departement des biostatistiques, domaine du Sart Tilam, B35, 4000 Liège, Belgium; European Drug Development Consulting, rue du Tambour, 4, 4540 Amay, Belgium; Emergency Department, CHR de la Citadelle, boulevard du 12eme de ligne, 1, 4000 Liège, Belgium

## Abstract

**Introduction:**

Identifying patients who need damage control resuscitation (DCR) early after trauma is pivotal for adequate management of their critical condition. Several trauma-scoring systems have been developed to identify such patients, but most of them are not simple enough to be used in prehospital settings in the early post-traumatic phase. The Trauma Induced Coagulopathy Clinical Score (TICCS) is an easy-to-measure and strictly clinical trauma score developed to meet this medical need.

**Methods:**

TICCS is a 3-item clinical score (range: 0 to 18) based on the assessment of general severity, blood pressure and extent of body injury and calculated by paramedics on-site for patients with severe trauma. This non-interventional prospective study was designed to assess the ability of TICCS to discern patients who need DCR. These patients were patients with early acute coagulopathy of trauma (EACT), haemorrhagic shock, massive transfusion and surgical or endovascular haemostasis during hospitalization. Diagnosis of EACT was assessed by both thromboelastometry and conventional coagulation tests.

**Results:**

During an 18-month period, 89 severe trauma patients admitted to the general emergency unit at our hospital were enrolled in the study, but 7 were excluded for protocol violations. Of the 82 remaining patients, 8 needed DCR and 74 did not. With receiver operating characteristic curve analysis, TICCS proved to be a powerful discriminant test (area under the curve = 0.98; 95% CI: 0.92 to 1.0). A cutoff of 10 on the TICCS scale provided the best balance between sensitivity (100%; 95% CI: 53.9 to 100) and specificity (95.9%; 95% CI: 88.2 to 99.2). The positive predictive value was 72.7%, and the negative predictive value was 100.0%.

**Conclusion:**

TICCS can be easily and rapidly measured by paramedics at the trauma site. In this study of blunt trauma patients, TICCS was able to discriminate between patients with and without need for DCR. TICCS on-site evaluation should allow initiation of optimal care immediately upon hospital admission of patients with severe trauma in need of DCR. However, a larger multicentre prospective study is needed for in-depth validation of TICCS.

**Trial registration:**

Clinicaltrials.gov ID: NCT02132208 (registered 6 May 2014)

## Introduction

Uncontrolled exsanguinating haemorrhage is the leading cause of death within the first 48 hours after severe trauma [1-3]. Almost 25% of major trauma patients present with a trauma-induced coagulopathy characterized by an initial haemorrhagic phenotype (early acute coagulopathy of trauma (EACT)) and a potential late procoagulant phenotype [4-6]. Previously thought to be the consequence of the old ‘lethal triad’ concept (hypothermia, dilution, coagulopathy), EACT is actually a far more complex phenomenon that occurs with a haemorrhagic phenotype in the early phase after trauma [4].

Initiating damage control resuscitation (DCR) as early as possible after severe trauma in patients with EACT is pivotal for patient survival [7]. This specific and aggressive therapeutic strategy and its components have been widely studied and debated [8-13], but one aspect remains essential for the patient’s outcome: The treatment has to be initiated as early as possible to be efficient [8]. DCR combines damage control surgery, permissive hypotension and early aggressive haemostatic resuscitation. However, it also implies surgical and transfusion resources available in trauma centres 24 hours per day, 7 days per week. For economic reasons, these resources cannot be offered immediately upon admission to emergency units of general hospitals, because a minimal delay is required for organization.

Identification of trauma patients who need DCR is a real challenge. The presence of EACT is strongly associated with the need for DCR. The results of recent studies suggest that standard coagulation tests such as the international normalized ratio (INR) or the activated partial thromboplastin time help in the detection of EACT, but these measures are time-consuming and probably lack relevance to guide the clinician in transfusion management. Thromboelastometric (TEM) assays may be better in identifying EACT and can rapidly bring clinically useful information [8,14,15]. However, the clinical predictive value of TEM has not been clearly established in this setting, and TEM probably underestimates the potential presence of early occult hyperfibrinolysis. All potential biological coagulation analyses, however, require that the patient has been admitted to the hospital, and they are time-consuming.

Several trauma scoring systems have been developed for stratification of the patient’s risk for the need for massive transfusion (MT) or the existence of EACT [16-24]. To be predictive, these scores generally include weighted and sophisticated systems, making them difficult to be used in routine practice. All of them, except the coagulopathy of severe trauma (COAST) score, require not only clinical data but also laboratory investigations or medical ultrasonic examinations [19], delaying their use at least a few minutes after hospital admission. Thus, they do not allow prehospital identification of patients who need DCR.

In Belgium, in the absence of trauma centres, trauma patients are referred to general hospitals. Thus, in our hospital (CHR de la Citadelle, Liège, Belgium), about 50 severe trauma patients are referred to our emergency unit each year, ‘diluted’ among more than 65,000 nontrauma cases. This ‘dilution’ prohibits maintaining the necessary organisation for immediate initiation of DCR and therefore preventing trauma patients with EACT to be optimally managed. In order to offer DCR to severe trauma patients with EACT at hospital admission, we developed the Trauma Induced Coagulopathy Clinical Score (TICCS), aiming to ‘flag’ trauma patients who need DCR at the site where the traumatic injury has taken place so that the hospital can take the necessary organisational steps before the patient’s arrival.

In contrast to currently available trauma scoring systems, TICCS was developed as an easy-to-use, strictly clinical score that can be calculated quickly by paramedics at the trauma site. It does not require any laboratory tests, X-rays or ultrasound information, as opposed to other trauma scores (for example, Trauma Associated Severe Haemorrhage (TASH), assessment of blood consumption (ABC), Prince of Wales Hospital (PWH) score, Schreiber score, Larsen score or Vandromme score). The three clinical components of the score were selected on the basis of practicability and known relationships to trauma severity and risk for active bleeding, namely: general severity of the trauma, blood pressure and extent of tissue injuries. In contrast to the COAST score, the prehospital body temperature was not considered as a relevant predictor. Further, there was no incorporation of difficult aspects, such as diagnosis of pelvic dislocation or abdominal bleeding. The TICCS system attributes a ‘score’ totalling between 0 and 18 points as described hereafter: (1) *General severity of the trauma*: 2 points are attributed if the patient is judged in critical condition and to be oriented to the resuscitation room (based on the general severity of the trauma: kinetics considerations, airway and breathing examinations, Glasgow Coma Scale) and 0 otherwise (that is, to be oriented to a regular emergency department room). (2) *Blood pressure*: 5 points are attributed if the prehospital systolic blood pressure is below 90 mmHg at least once and 0 if it stayed continuously above 90 mmHg. (3) *Extent of tissue injuries*: 11 points are attributed for the extent of body injury, depending on the presence of a significant injury, as follows: 1 point for the head and neck region, 1 point for each four extremities, 2 points for the torso region, 2 points for the abdominal region and 2 points for the pelvic region (Table 1). Paramedics and prehospital doctors were trained how to calculate TICCS.

**Table 1:** **Definition and scoring system of the Trauma Induced Coagulopathy Clinical Score (TICCS)**

**Criteria**	**Number of points attributed**
General severity	
Critical (to be admitted in resuscitation room)	2
Non critical (regular ED room)	0
Blood pressure	
SBP below 90 mmHg at least once	5
SBP always above 90 mmHg	0
Extent of significant injuries	
Head and neck	1
Left upper extremity	1
Right upper extremity	1
Left lower extremity	1
Right lower extremity	1
Torso	2
Abdomen	2
Pelvis	2
Total possible score	0 to 18

^a^ED, Emergency department; SBP, Systolic blood pressure.

The present study was conducted to evaluate the efficacy of TICCS to discriminate major trauma patients requiring DCR from those who do not.

## Material and methods

This prospective, single-centre, non-interventional, noncontrolled, open clinical study was submitted to and approved by the Ethics Committee of our hospital (CHR de la Citadelle, Liège, Belgium). Considering the non-interventional nature of the study and the impossibility of obtaining the patients’ informed consent before their enrolment in the study, the study was authorized by the concerned institutional ethics committee without informed consent. The study started in January 2012 and ended in June 2013.

For all patients, the TICCS was calculated and recorded before any measurement of hospital severity parameters. Complementary to TICCS, the Injury Severity Score, ABC and TASH scores were also measured in the emergency unit [19,20]. The presence of EACT was assessed by thromboelastometry using ROTEM (Tem Innovations, Munich, Germany) at the latest 30 minutes after patient admission and by standard coagulation tests (INR, fibrinogen). Coagulopathy was defined as the presence of a significant abnormality (more than 20%) for at least one of the following parameters in ROTEM: clotting time, clot formation time, maximum clot firmness (MCF) and maximum lysis for extrinsic thromboelastometry or MCF for fibrinogen thromboelastometry or as INR >1.3 at admission and/or after 3 hours, or fibrinogen <1.5 g/L at admission and/or 3 hours later. Coagulation tests were carried out by laboratory technicians not aware of the TICCS value.

Haemorrhagic shock was assessed by the attending physician at the time of hospital admission on the basis of persistent hypotension due to proven active bleeding. The transfusion of more than 4 RBC units and more than 3 fresh frozen plasma (FFP) units within the first hour of care was defined as a MT. The global need for transfusion within the first 24 hours was also recorded. Surgical or endovascular haemostatic procedures were recorded throughout hospitalization. Patients who were dying because of a confirmed haemorrhagic shock at the early phase of care in the resuscitation room before being able to benefit from surgery were classified as needing emergent surgical haemostasis and MT. The study patients were categorized in two groups. ‘Severe’ patients (in need of DCR) were those satisfying all three clinical criteria (diagnosis of haemorrhagic shock associated with MT, use of surgical or endovascular haemostatic procedure) and the laboratory criterion (EACT). By contrast, ‘nonsevere’ patients (not in need of DCR) were defined as patients who did not fulfil at least one of the four criteria stated above.

Quantitative variables were summarized as median and interquartile range (IQR) as well as range, and frequency tables were used for categorical findings. Group comparisons were done by applying the Kruskal-Wallis test for continuous variables and the χ^2^ test (or Fisher’s exact test) for categorical variables. The cutoff value for TICCS was obtained by ROC curve analysis based on the severity of the patients’ condition. Each trauma score was characterized by its sensitivity, specificity, positive and negative predictive values (PPV and NPV) and area under the ROC curve (AUC) with 95% confidence intervals (95% CI). The results were considered significant at the 5% critical level (*P* < 0.05). Calculations were performed with the SAS version 9.3 for Windows statistical software package (SAS Institute, Cary, NC, USA).

## Results

Between January 2012 and June 2013, about 100,000 patients were admitted to the emergency unit of our hospital. Among them, 89 (0.09%) had severe trauma and were enrolled in the study. Seven patients were excluded from the analysis, however, because of protocol violations or complete absence of data. Thus, the statistical analysis was based on 82 study patients. Their characteristics are described in Table 2.

**Table 2:** **Characteristics of the study population (** ***N*** **= 82)** ^**a**^

**Variable**	**Category**	***N***	***n*** **(%)**	**Median (IQR)**	**Range**
Sex		82			
	F		18 (22.0)		
	M		64 (78.1)		
Age (yr)		82		34.5 (23.0 to 45.0)	14.0 to 82.0
ISS		82		13.0 (9.0 to 22.0)	4.0 to 66.0
EXTEM		50			
	Hypocoagulability		8 (16.0)		
	Normal		42 (84.0)		
FIBTEM		50			
	Hypocoagulability		13 (26.0)		
	Normal		37 (74.0)		
INR at admission		81		1.1 (1.0 to 1.1)	1.0 to 2.9
INR after 3 hr		69		1.1 (1.1 to 1.2)	1.0 to 2.1
Fibrinogen at admission (g/L)		81		2.7 (2.1 to 3.2)	0.4 to 6.4
Fibrinogen after 3 hr (g/L)		68		3.0 (1.8 to 3.7)	0.7 to 8.5
Hb at admission (g/L)		81		13.9 (12.7 to 15.0)	6.7 to 16.7
Admission base excess		82		−1.0 (−4.0 to 0.0)	−16.0 to 3.0
Haemorrhagic shock		82			
	No		71 (86.6)		
	Yes		11 (13.4)		
Emergent surgical haemostasis		82			
	No		71 (86.6)		
	Yes		11 (13.4)		
SBP at admission (mmHg)		82		130 (120 to 140)	60.0 to 200.0
Glasgow Coma Scale		82		11.0 (3.0 to 15.0)	3.0 to 15.0
24-hr survival			75 (91.5)		
30-day survival			72 (87.8)		

^a^EXTEM, Extrinsic thromboelastometry; FIBTEM, Fibrinogen thromboelastometry; Hb, Haemoglobin; INR, International normalized ratio; ISS, Injury Severity Score; SBP, Systolic blood pressure.

**Figure 1:**
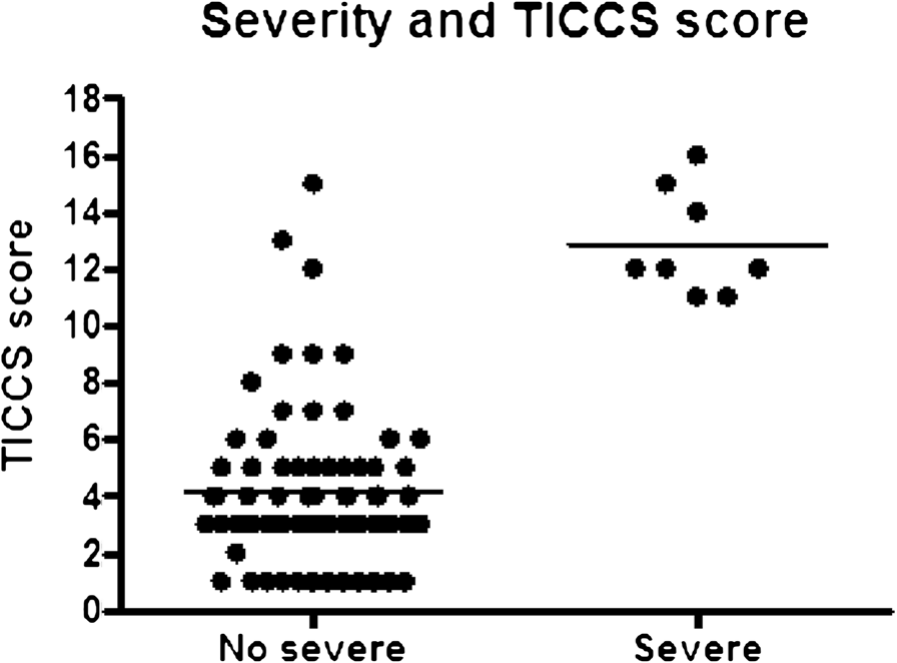
**TICCS values in both subgroups.** TICCS, Trauma Induced Coagulopathy Clinical Score.

**Figure 2:**
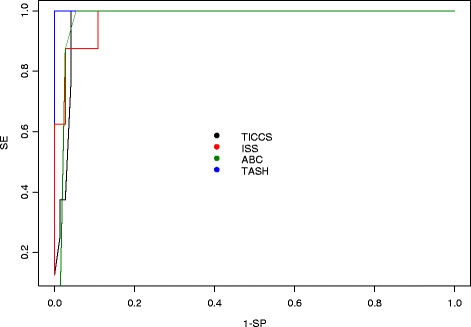
**Area under the receiver operating characteristic curve of the measured scores.** ABC, Assessment of blood consumption; ISS, Injury Severity Score; SE, Sensitivity; SP, Specificity; TASH, Trauma Associated Severe Haemorrhage; TICCS, Trauma Induced Coagulopathy Clinical Score.

Among the 82 patients, 74 were classified as ‘nonsevere’ and 8 as ‘severe’ (in need of DCR). The median (IQR) TICCS was 3 [3-5] for ‘nonsevere’ patients and 12 [12-15] for ‘severe’ patients (Figure 1). The two groups differed significantly (*P* = 0.0011). ROC curve analysis showed that TICCS was able to discriminate between severe and nonsevere patients with an AUC of 0.98 (95% CI: 0.92 to 1.0). Further, a TICCS cutoff value of 10 yielded the best trade-off between true positives and false positives. Table 3 displays the characteristics of the study patients according to TICCS <10 and TICCS ≥10. The corresponding sensitivity and specificity of TICCS were 100% (95% CI: 53.9 to 100) and 95.9% (95% CI: 88.2 to 99.2), respectively, and the PPV and NPV were equal to 72.7% (95% CI: 43.3 to 68.6) and 100% (95% CI: 94.7 to 100), respectively. These figures are superior to those obtained for the other scores (see Table 4 and Figure 2). The three false-positive patients (TICCS ≥10 but ‘nonsevere’) had EACT but did not meet all three clinical criteria (Table 5).

**Table 3:** **Patients’ characteristics by Trauma Induced Coagulopathy Clinical Score groups** ^**a**^

		**TICCS <10**		**TICCS ≥10**		
**Variables**	**Category**	***N***	**Median (IQR)** ***n*** **(%)**	***N***	**Median (IQR) or** ***n*** **(%)**	***P*** **-values**
Sex		71		11		0.44
	F		17 (23.9)		1 (9.1)	
	M		54 (76.1)		10 (90.9)	
Age (yr)		71	34.0 (23.0 to 45.0)	11	35.0 (18.0 to 43.0)	0.94
EXTEM		43		7		<0.0001
	Hypocoagulability		1 (2.3)		7 (100.0)	
	Normal		42 (97.7)		0 (0.0)	
FIBTEM		43		7		<0.0001
	Hypocoagulability		6 (14.0)		7 (100.0)	
	Normal		37 (86.0)		0 (0.0)	
INR at admission		71	1.0 (1.0 to 1.1)	10	1.3 (1.1 to 1.4)	0.0048
INR after 3 hr		60	1.1 (1.1 to 1.2)	9	1.4 (1.3 to 1.5)	0.0002
Fibrinogen at admission (g/L)		71	2.8 (2.2 to 3.3)	10	1.7 (1.3 to 2.0)	0.0041
Fibrinogen after 3 hr (g/L)		59	3.2 (2.3 to 3.8)	9	1.5 (1.3 to 1.5)	<0.0001
Hb at admission (g/L)		71	13.9 (13.1 to 15.1)	10	12.4 (9.4 to 14.0)	0.026
Haemorrhagic shock		71		11		<0.0001
	No		70 (98.6)		1 (9.1)	
	Yes		1 (1.4)		10 (90.9)	
Emergent surgical haemostasis		71		11		<0.0001
	No		67 (94.4)		4 (36.4)	
	Yes		4 (5.6)		7 (63.6)	
RBCs transfused day 1 (U)		71	0.0 (0.0 to 1.0)	11	6.0 (3.0 to 12.0)	<0.0001
FFP transfused day 1 (U)		71	0.0 (0.0 to 0.0)	11	4.0 (2.0 to 8.0)	<0.0001
Platelets transfused		71		11		0.0002
day 1	No		71 (100.0)		7 (63.6)	
	Yes		0 (0.0)		4 (36.4)	
24-hr survival		71		11		<0.0001
	No		1 (1.4)		6 (54.6)	
	Yes		70 (98.6)		5 (45.4)	
30-day survival		71		11		0.0002
	No		4 (5.6)		6 (54.6)	
	Yes		67 (94.4)		5 (45.4)	

^a^EXTEM, Extrinsic thromboelastometry; FFP, Fresh frozen plasma; FIBTEM, Fibrinogen thromboelastometry; Hb, Haemoglobin; INR, International normalized ratio; RBCs, Red blood cells; TICCS, Trauma Induced Coagulopathy Clinical Score.

**Table 4:** **Comparison of sensitivity, specificity, positive and negative predictive value and area under the receiver operating characteristic curve of the various trauma scores measured during the study** ^**a**^

**Score cutoff**	**SE (%)**	**SP (%)**	**PPV (%)**	**NPV (%)**	**AUC**
TICCS ≥10	100.0	95.9	72.7	100.0	0.98
ISS ≥25	86.5	100.0	44.4	100.0	0.93
ABC ≥2	100.0	94.6	66.7	100.0	0.97
TASH ≥16	100.0	62.5	100.0	96.1	0.81

^a^ABC, Assessment of blood consumption; AUC, Area under the receiver operating characteristic curve; ISS, Injury Severity Score; NPV, Negative predictive value; PPV, Positive predictive value; SE, Sensitivity; SP, Specificity; TASH, Trauma Associated Severe Haemorrhage; TICCS, Trauma Induced Coagulopathy Clinical Score.

**Table 5:** **Characteristics of the three ‘nonsevere’ patients with Trauma Induced Coagulopathy Clinical Scores ≥10** ^**a**^

**Patient no.**	**Haemorrhagic shock**	**Emergent surgical haemostasis**	**Massive transfusions**	**EACT**
1	No	No	No	Yes
2	Yes	Yes	No	Yes
3	Yes	No	No	Yes

^a^EACT, Early acute coagulopathy of trauma; TICCS, Trauma Induced Coagulopathy Clinical Score.

## Discussion

Early identification of trauma patients in need of DCR is essential to provide adequate treatment upon hospital admission and to have a major impact on their outcomes [25]. By contrast, initiation of DCR in patients who do not require this aggressive therapy may negatively affect their survival [26]. The on-site flagging of severe trauma patients on the basis of TICCS has potential to be beneficial for general emergency units that are not expected to be ready for this rare situation 24 hours per day, 7 days per week. It should also be useful for high-performing trauma centres to identify such patients and transfer them immediately to the resuscitation room upon admission. This would in turn avoid the use of colloids or lead to initiation of prehospital administration of blood products and/or haemostatic agents and MT protocols. Finally, TICCS could be useful for triaging patients in collective trauma situations. It should be noted that, as our trauma population consisted essentially of blunt trauma patients, at present the use of TICCS should be restricted to such patients.

Although based on a limited sample size, the study evidenced TICCS as a potential candidate for on-site identification of severe trauma patients in need of DCR. Larger multicentre studies are needed to confirm these preliminary results.

## Conclusions

The results of the present clinical study confirm that TICCS, an easy and quick severe trauma scoring system measured on site by paramedics, has the ability to identify patients in need of DCR. Early prehospital ‘flagging’ of those patients should allow general emergency units to mobilize the specific resources requested to offer high-quality DCR for the limited number of patients who need it (impact on cost-effectiveness of patients’ support) and to shorten the time between injury and DCR initiation (impact on patients’ survival). The MT protocol in place at our hospital [27] can be activated in due time, allowing thawing the necessary FFP units and preparing the packed RBC and platelet units before patient admission. Surgeons and interventional radiologists will also be ready for interventions for the patients. The TICCS differs from the other scores, such as ABC, TASH, Vandromme or PWH, by being purely clinical and easy to compute by paramedics at the site of injury. Our study results also show that the diagnostic ability of TICCS was superior to ABC and TASH. Besides its interest for general emergency units, the TICCS could also prove to be a useful tool for high-quality trauma centres and trauma systems in terms of prehospital triage and management, especially in the setting of prehospital bleeding management and transfusion of blood products and use of haemostatic agents or crystalloids and colloids.

## Key messages

- Prehospital identification of patients with severe trauma who need DCR is a prerequisite for correct early management of such high-risk patients.
- Most existing trauma scoring systems cannot be calculated on site before a patient’s admission to the emergency unit.
- TICCS can potentially fulfil this medical need and could have a significant impact on patient mortality.
- TICCS is an easily and rapidly computed score that can be used by paramedics at the trauma site.
- For blunt trauma patients, TICCS demonstrates a clear ability to discriminate patients with vs without a need for DCR.
- Further validation of TICCS is needed to confirm the presently reported results, such as by means of larger, multicentre, prospective studies.

## Abbreviations

ABC: Assessment of blood consumption
AUC: Area under the receiver operating characteristic curve
COAST: Coagulopathy of severe trauma
DCR: Damage control resuscitation
EACT: Early acute coagulopathy of trauma
EXTEM: Extrinsic thromboelastometry
FFP: Fresh frozen plasma
FIBTEM: Fibrinogen thromboelastometry
INR: International normalized ratio
ISS: Injury Severity Score
MCF: Maximum clot firmness
MT: Massive transfusion
NPV: Negative predictive value
PPV: Positive predictive value
PWH: Prince of Wales Hospital
RBC: Red blood cell
ROC: Receiver operating characteristic
TASH: Trauma Associated Severe Haemorrhage
TICCS: Trauma Induced Coagulopathy Clinical Score
